# Artificial Intelligence in Stroke Care: A Narrative Review of Diagnostic, Predictive, and Workflow Applications

**DOI:** 10.7759/cureus.93430

**Published:** 2025-09-28

**Authors:** Vasant T Heeralal, Saiesha E Chadee, Benjamin Ilyaev, Rafael Ilyaev, Stella Ilyayeva

**Affiliations:** 1 Medicine, The University of the West Indies, St. Augustine, TTO; 2 Medicine, Hofstra University, Hempstead, USA; 3 Medicine, New York Institute of Technology, Old Westbury, USA; 4 Endocrinology and Diabetes, Atlantic Endocrinology and Diabetes, Queens, USA

**Keywords:** artificial intelligence, neurology and critical care, neurosurgery, radiology, stroke care

## Abstract

Artificial intelligence (AI) has emerged as a transformative force in stroke care, with increasing integration into diagnostic, predictive, and operational domains. This narrative review synthesizes the applications of AI in acute stroke management, drawing on peer-reviewed literature published between 2015 and 2024. A structured search of PubMed, Google Scholar, Semantic Scholar, National Center for Biotechnology Information (NCBI), and Litmaps identified 300 records, of which 46 met predefined criteria. Eligible studies were peer-reviewed, in English, and focused on ischemic or hemorrhagic stroke with reported clinical, operational, or system-level outcomes; studies limited to algorithm development or non-original data were excluded. “Real-world” contexts were defined as those involving implemented or externally validated tools, while international studies were included only when their findings were directly applicable to U.S. practice. This review was conducted narratively, organized by diagnostic, predictive, and workflow domains.

In diagnostic imaging, AI platforms have demonstrated efficacy in detecting large vessel occlusions, hemorrhage, and perfusion deficits, expediting triage in time-critical scenarios. Predictive modeling tools support outcome forecasting and hemorrhagic risk stratification, while workflow applications such as AI-powered coordination platforms improve communication, accelerate decision-making, and reduce treatment delays. Some tools, including RapidAI and Viz.ai, have undergone multicenter validation, but most remain in early or proof-of-concept phases. Ethical concerns persist, particularly regarding dataset bias, lack of interpretability, and uneven access to advanced imaging infrastructure. Cost-effectiveness analyses remain sparse, leaving uncertainty about scalability in resource-limited settings.

Collectively, these tools function not as autonomous decision-makers but as augmentative supports that reinforce clinical judgment and operational efficiency. The current evidence base highlights gaps that future research must address: multicenter prospective validation, standardized cost-effectiveness studies, equity-focused deployment, and explainability frameworks. Despite these limitations, AI is increasingly positioned as a scaffolding mechanism within stroke systems, enhancing rather than replacing the work of clinicians. Its evolution reflects a shift from proof-of-concept innovation to infrastructural augmentation, with its future impact contingent on rigorous validation, ethical design, and system-level alignment.

## Introduction and background

Stroke as a clinical and operational emergency

Recent projections indicate an increase in age-standardized ischemic stroke incidence to 89.32 per 100,000 by 2030, despite a decrease in global death and disability-adjusted life year (DALY) rates [1]. A DALY represents a composite measure of disease burden, capturing both years of life lost due to premature mortality (YLL) and years lived with disability (YLD). Because these values are age-adjusted, they allow comparison across populations and provide a clearer picture of stroke's long-term societal impact [1]. In the United States, 7.09 million people were living with stroke in 2019, with ischemic strokes accounting for 82.7% of cases [2]. Hemorrhagic strokes, while less common, have shown a steeper rise in mortality and remain an important contributor to poor outcomes [2]. Geographic disparities also shape this burden, with younger adults in Southern and Mid-Western states experiencing rising incidence rates [3].

Timely intervention is central to effective stroke care because of the sharply limited therapeutic window. The phrase "time is brain" reflects the irreversible loss of nearly 1.9 million neurons per minute during untreated ischemia, yet national data reveal persistent delays in patient arrival at emergency departments [4]. Studies report that prehospital times have not improved meaningfully in over two decades despite advances in in-hospital door-to-needle metrics [4,5]. The contributing factors include poor symptom recognition, delayed emergency medical services (EMS) activation, and logistical barriers, particularly in rural and underserved communities [5]. Analyses of prehospital performance show that rural EMS systems experience longer delays compared with urban ones, driven by differences in training, stroke scale use, and hospital prenotification [5]. These prehospital barriers hinder access to reperfusion therapies such as intravenous thrombolysis and mechanical thrombectomy, both of which can dramatically alter patient outcomes when delivered on time [6].

Operational complexity further magnifies the challenge, as ischemic and hemorrhagic strokes follow different pathways but both impose heavy demands on hospital systems. Large vessel occlusions may require immediate endovascular intervention, while hemorrhagic events often necessitate intensive monitoring and neurosurgical capacity [7]. Despite improvements in protocols, fewer than 5% of eligible patients worldwide receive thrombolysis, and stroke unit access remains severely restricted in low-resource settings [6,8]. Even in high-income countries, workflow inefficiencies persist, from EMS prenotification gaps to imaging bottlenecks, delaying treatment initiation and straining frontline staff [9].

Beyond geography, inequities are also driven by socioeconomic and racial factors. Black and Hispanic populations in the United States experience higher incidence rates, greater stroke severity, and worse outcomes compared with White patients [9]. Post-acute recovery is shaped by comorbidities such as atrial fibrillation and diabetes, which complicate adherence to evidence-based pathways and increase the risk of recurrent events [10]. Taken together, these clinical, social, and operational barriers reveal stroke as not only a time-critical emergency but also a system-wide logistical challenge. Addressing these inefficiencies provides the foundation for introducing artificial intelligence, which has the potential to streamline recognition, standardize triage, and bridge gaps in timely care delivery.

Standard of care and clinical workflows

The clinical management of stroke, whether ischemic or hemorrhagic, is governed by structured protocols intended to minimize long-term disability and improve survival. Guidelines from major associations emphasize early symptom recognition, swift imaging, and time-sensitive therapeutic decision-making, regardless of stroke type [11]. However, disparities in implementation exist across facilities due to inconsistent staff training, resource availability, and clarity in care pathways [12]. These issues can affect critical decisions such as when to administer thrombolytics in ischemic stroke or manage blood pressure and coagulopathy in hemorrhagic events. Even when best practices are outlined, operational realities often determine how closely standard protocols are followed in real-world settings.

Clinical workflows across emergency and inpatient settings are frequently interrupted by delays in stroke recognition, imaging, and treatment initiation. In-hospital ischemic strokes are consistently associated with longer recognition-to-imaging intervals and delayed treatment initiation compared to community-onset cases. These delays contribute to lower rates of reperfusion therapy and poorer functional outcomes across multiple care settings [13]. Registry-based analyses from national stroke programs further demonstrate that diagnostic uncertainty, treatment hesitancy, and the complexity of managing comorbid conditions all contribute to extended door-to-needle times. These systemic inefficiencies directly increase the risk of adverse events and undermine the effectiveness of evidence-based interventions [14].

Workflow efficiency also hinges on minimizing interfacility transfers and diagnostic redundancies that can compromise timely stroke intervention. Patients who are directly admitted to comprehensive stroke centers achieve better outcomes than those transferred mid-care; this underscores how structural logistics shape patient trajectories [15]. Additionally, imaging complexity, especially with multimodal CT or MRI, has been shown to introduce avoidable delays when protocols are not streamlined [16]. While advanced imaging is clinically useful, its operational impact must be accounted for in workflow design. A high-functioning stroke system integrates both standardization and adaptability to ensure equitable treatment access across all stroke types and settings.

Rationale for AI in stroke care

Stroke management involves high-stakes, time-sensitive decision-making where variability in care delivery often leads to suboptimal outcomes. Standard clinical protocols rely on rapid imaging interpretation and multidisciplinary coordination, yet bottlenecks in these processes remain widespread [17]. Studies highlight inconsistencies in stroke assessment accuracy, delays in imaging-to-intervention timelines, and diagnostic challenges across both ischemic and hemorrhagic presentations [18,19]. These inefficiencies underscore the need for decision support at critical junctures. In practice, variability is especially pronounced in determining eligibility for intravenous thrombolysis, classifying hemorrhagic subtypes, and prioritizing patients for endovascular thrombectomy. AI platforms are designed to standardize these assessments, reduce diagnostic subjectivity, and shorten the time required for decisive action [17,18].

Operationally, the rising demand for stroke services has strained emergency and radiology departments, exposing system-level gaps that AI may address. Physician fatigue, time constraints, and increasing caseloads contribute to delayed or inconsistent decision-making [18,20]. The supporting evidence is heterogeneous: the majority of published studies remain retrospective or proof-of-concept models, a smaller number are prospective observational cohorts, and only a few have undergone multicenter validation [17,21,22]. For example, a recent meta-analysis of MRI-based AI detection reported pooled sensitivities and specificities above 90 percent, but noted that only one Conformité Européenne (CE)-approved algorithm exists and no Food and Drug Administration (FDA)-approved models have yet reached widespread clinical deployment [20]. Conversely, workflow-focused platforms have demonstrated measurable gains in real-world practice: implementation of an AI coordination tool within a hub-and-spoke system was associated with significant reductions in inter-facility transfer times and shorter length of stay [19], while AI-assisted CT detection models improved physician performance in stroke recognition, supporting faster and more consistent early decision-making [21]. Together, these findings indicate that while much of the field remains in the early stages, there is growing evidence that AI can improve alignment with best-practice benchmarks.

Despite promising performance reports, implementation raises important ethical and regulatory considerations. Many models remain opaque, which limits transparency in high-stakes decisions; concerns about dataset bias, generalizability, and accountability are repeatedly noted in systematic reviews and critical appraisals of current AI stroke tools [17,22]. Regulatory expectations for “software as a medical device” emphasize validation, human oversight, and post-market performance monitoring, yet few stroke AI systems have met evidence standards consistent with broad clinical adoption, reflecting the early maturity of the field [17,22]. These mechanisms are essential to ensure that AI augments rather than undermines clinical judgment.

Economic and scalability considerations are also critical. Formal health-economic evaluations specific to stroke AI remain limited in the published literature, and existing reviews emphasize persistent gaps in cost and implementation data [17]. Scalability in resource-limited settings is further constrained by infrastructure requirements such as timely imaging access, reliable connectivity, and trained personnel, which mirror broader global access barriers to acute stroke interventions [6]. These realities argue for staged adoption and context-specific evaluation of clinical and operational impact before large-scale roll-out.

The connection between workflow bottlenecks and AI-enabled process gains has begun to appear in real-world reports. In hub-and-spoke networks, AI-driven coordination platforms have reduced inter-facility transfer times and hospital length of stay, highlighting their potential to improve system efficiency [19]. Complementary evidence shows that AI assistance can enhance physician accuracy in stroke detection on CT imaging, although effects on downstream treatment intervals require further study [21]. Meta-analytic work in MRI further underscores high technical accuracy while emphasizing the need for prospective usability and clinical utility trials before routine deployment [20]. Overall, these findings support a pragmatic view of AI as a means to standardize high-impact decision points and compress time-sensitive steps in the stroke pathway as evidence matures. The rationale for AI in stroke care therefore lies not in replacing clinicians, but in reinforcing reliability, reducing variability, and improving equity in a domain where time and accuracy are paramount.

AI in diagnostic imaging for stroke

Diagnostic imaging is foundational in acute stroke care, guiding decisions about hemorrhage exclusion, large vessel occlusion (LVO) detection, and tissue viability. Modalities such as non-contrast CT (NCCT), CT angiography (CTA), CT perfusion (CTP), and MRI provide complementary insights, but interpretation remains time-sensitive and cognitively demanding. These pressures have fueled the adoption of AI tools to support high-stakes diagnostic tasks. Early work demonstrated that automated CTA analysis could detect intracranial LVO with strong performance compared to neuroradiologists, establishing proof of concept for AI in vascular triage [23]. Parallel advances in NCCT interpretation have enabled detection of acute or subacute hemorrhage, with confidence scores that quantify uncertainty and highlight potential false-positives or -negatives [24]. In addition, AI-calculated Alberta Stroke Program Early CT Scores (ASPECTS) have been evaluated for reliability, showing good concordance with radiologists while reducing inter-reader variability [25]. Together, these studies underscore the rationale for AI adoption in imaging, not as a substitute for clinical judgment but as a mechanism to standardize performance in fast-paced environments.

Beyond image interpretation, AI has been deployed to streamline communication and team coordination. Platforms such as Viz.ai and RapidAI integrate with picture archiving and communication systems (PACS) and electronic health records (EHRs) to deliver automated alerts, mobile notifications, and triage dashboards [26,27]. The Viz.ai Utilization to Limit Delays in Acute Stroke Treatment (VALIDATE) study showed that response times improved with Viz.ai, and that sustained benefit required alignment across departments and reliable infrastructure [26]. Similarly, implementation reports documented measurable accelerations in team activation but emphasized that realized gains depended on consistent workflow adoption and organizational readiness [27].

Several studies have examined efficiency metrics after AI integration. Implementation has been linked to shorter door-to-needle and door-to-puncture times by streamlining triage and escalation [28,29], as well as reduced interfacility transfer delays in hub-and-spoke networks [29]. A multi-institutional analysis further demonstrated significantly lower door-to-puncture intervals following deployment of AI-enabled workflows compared with pre-AI baselines [30]. These findings suggest that AI's value extends beyond detection alone, functioning as an operational adjunct that compresses treatment windows in routine practice.

Performance accuracy has also been validated. In one evaluation, an AI algorithm for CTA achieved sensitivity of 0.82 and accuracy of 0.89 in LVO detection, supporting its use in rapid triage [31]. Automated ASPECTS software has likewise improved reader agreement and diagnostic consistency across experience levels [32]. Limitations remain. Interobserver reliability for NCCT ASPECTS, even with AI support, is not eliminated [33], and many tools lack external validation across diverse populations and scanner environments, which limits generalizability [34]. False classifications carry meaningful clinical consequences: missed LVOs may delay thrombectomy, while spurious hemorrhage detection may inappropriately preclude reperfusion [24]. These realities emphasize that AI should be viewed as augmentative, helping accelerate communication, improve standardization, and reduce cognitive burden, while preserving clinician oversight [25,34].

AI predictive modeling in stroke care

Prognostic uncertainty is a recurring challenge in stroke, where early decisions often hinge on anticipated clinical courses. AI-based predictive modeling seeks to improve individualized forecasts of functional recovery, mortality, and hemorrhagic risk, complementing clinician judgment rather than replacing it [18,35,36]. Most published models have been developed and evaluated in retrospective cohorts, which supports feasibility but limits causal interpretation and generalizability [35,36].

These models draw on routinely available inputs. Clinical scales such as the National Institutes of Health Stroke Scale (NIHSS), a 15-item standardized assessment of neurological deficit, and imaging scores such as ASPECTS, a 10-point CT measure of early ischemic change in middle cerebral artery territories, commonly anchor feature sets alongside laboratory markers like glucose and creatinine [37,38]. Model architectures vary, including gradient boosting, convolutional neural networks, and ensemble approaches. Performance is typically reported with area under the curve and mean absolute error (MAE), where MAE reflects the average absolute difference between predicted and observed outcomes. To streamline development and improve interpretability, automated machine learning (AutoML) can automate model selection while SHapley Additive exPlanations (SHAP) provide case-level and feature-level attribution [39].

Applications span acute and subacute decision points. Early after admission, individualized prediction of discharge NIHSS within the first day has been shown using an XGBoost framework, supporting rapid risk stratification and care planning [37]. For patients considered for or treated with thrombolysis, machine learning models have been used to estimate the risk of hemorrhagic transformation, with meta-analytic evidence that modern algorithms such as XGBoost and artificial neural networks can improve risk stratification, while also calling for standardized reporting and stronger prospective designs [39]. The clinical implications of error are material: over-prediction of hemorrhage risk may deny eligible patients time-sensitive therapy, whereas under-prediction may expose patients to avoidable bleeding complications [39,40].

Comparative work suggests that machine learning can match or exceed conventional approaches in some settings, although results are mixed and context dependent. Studies comparing machine learning with logistic regression have reported similar or modestly higher discrimination, with reliability advantages in certain cohorts [35,41]. Prospective comparisons indicate that while machine learning may show higher area under the curve, clinical utility depends on calibration, transparency, and workflow fit [41]. In parallel, external validation using independent cohorts and imaging biomarkers has been emphasized as a prerequisite for broader adoption [42,43].

Methodological and implementation constraints persist. Retrospective designs and single-center datasets increase the risks of overfitting, missing data bias, and class imbalance if not explicitly managed and transparently reported [35]. Reviews have also underscored ethical and regulatory considerations, including the need for explainability in high-stakes use, clear oversight structures, and alignment with clinical decision support standards [18]. Evidence on cost-effectiveness and practical integration into existing systems remains limited, which makes staged adoption, calibration monitoring, and prospective, multicenter validation important targets for future work [18,39,42].

AI in workflow optimization for stroke care

Stroke outcomes are determined not only by clinical acuity but by how effectively systems function across the entire care continuum. From symptom onset to definitive treatment, stroke pathways are repeatedly disrupted by triage delays, paging inefficiencies, and variable staffing, especially during off-hours. These workflow failures are not incidental; they represent structural liabilities that delay intervention and diminish outcomes. In response, artificial intelligence has emerged as a system-level coordination layer, designed to align multidisciplinary teams, automate prioritization, and reduce decision latency [30,35]. Its value lies in enabling faster care delivery without demanding additional cognitive or staffing resources, reframing workflow as a modifiable determinant of stroke recovery [44].

Yet, advanced imaging can itself introduce delays. One analysis reported that door-to-needle times were higher in patients undergoing CTA before thrombolysis, with a median of 60 minutes compared to 51.5 minutes with NCCT and 48 minutes with duplex ultrasonography [16]. These trade-offs emphasize that while imaging expands diagnostic certainty, it may lengthen treatment initiation unless coupled with workflow optimization strategies such as AI-supported triage.

AI platforms such as the Viz.ai stroke triage platform (Viz.ai, Inc., San Francisco, CA, USA), RapidAI (RapidAI, Inc., San Mateo, CA, USA), and Brainomix e-Stroke (Brainomix Ltd., Oxford, UK) have redefined coordination by integrating imaging, alerts, and communication into a unified operational stream. Unlike tools focused solely on diagnostic interpretation, these systems embed within picture archiving and communication systems (PACS), electronic health records (EHRs), and EMS interfaces to trigger automated notifications and mobilize teams in parallel [27,31]. PACS primarily manages and stores medical images, while EHRs provide a broader repository of patient data, including clinical notes, laboratory results, and medication records. By linking these systems with real-time dashboards, mobile alerts, and prioritization logic, AI replaces fragmented paging chains with synchronized team activation. The result is not just faster escalation, but a more consistent and scalable response architecture that remains stable regardless of hospital size, staffing model, or time of day [45].

Clinical deployments of AI-coordinated workflows have yielded measurable performance improvements. In a multicenter evaluation, CTA-to-door-in transfer intervals were reduced by an average of 22.5 minutes when AI was integrated into a hub-and-spoke model [19]. Another analysis of Viz.ai reported a 39% improvement in workflow efficiency for direct-arriving LVO cases during off-hours [27]. Abstract evidence also highlights reductions in door-to-needle and door-to-puncture intervals following AI-supported coordination [28]. Additional studies confirmed that door-to-puncture times decreased by 15 minutes overall, with interhospital transfer patients experiencing a 37-minute reduction in transfer duration [29]. In a broader pre-post analysis, transferred patients had median door-to-puncture times of 142 minutes versus 169 minutes before AI deployment [30]. Complementary findings from a primary stroke center showed shorter door-to-needle times (44 → 42 minutes) and CT-to-groin puncture times (174 → 145 minutes) after the adoption of AI-decision support [45]. These improvements underscore AI's ability to accelerate triage-to-treatment processes, reduce unwarranted variation, and improve consistency in high-acuity settings.

Beyond operational efficiency, AI workflow tools address long-standing equity gaps in stroke care access. By supporting telestroke networks and enabling hub-and-spoke coordination, these platforms bridge disparities between urban and rural institutions and between high- and low-volume centers [44,46]. Automated triage ensures that treatment eligibility is not delayed by geography or institutional capacity. As stroke systems evolve, AI emerges as a form of adaptive infrastructure capable of reinforcing communication, compressing time, and reducing fragmentation at scale. Its role is not to replace clinicians but to ensure that the right teams are in the right place at the right time, every time.

## Review

Methodology

This narrative review was conducted to examine the clinical and operational impact of AI in acute stroke care. We aimed to capture the most relevant literature across diagnostic imaging, predictive modeling, and workflow optimization domains. Our team performed a structured search across PubMed, Google Scholar, National Center for Biotechnology Information (NCBI), Semantic Scholar, and Litmaps, focusing on peer-reviewed articles published between January 2015 and March 2024. Combinations of keywords included "stroke," "artificial intelligence," "machine learning," "clinical decision support," "diagnostic imaging," "predictive modeling," and "workflow coordination." Additional articles were identified through citation tracking and thematic clustering of related topics.

All retrieved studies were screened in multiple stages; those meeting preliminary criteria were stored and organized in Zotero (Corporation for Digital Scholarship, Vienna, VA, USA), where each was assigned, tags corresponding to the key domains of the review. Four reviewers independently screened the studies, and final inclusion was determined by consensus discussion. When disagreements occurred, they were resolved through group deliberation until agreement was reached.

Selection emphasized methodological quality, which we defined as peer-review status, clear study design, and reporting of clinical or operational outcomes with standard performance metrics such as sensitivity, specificity, or AUC. Articles were further assessed for their applicability to U.S. stroke care, with international studies included if their infrastructure (e.g., CT, CTA, reperfusion therapy, workflow protocols) aligned with U.S. practice. Studies addressing more than one functional domain were categorized according to their primary objective and outcomes.

To synthesize the findings, each study was reviewed in full and interpreted in the context of its clinical function, integration strategy, and reported outcomes. Particular attention was paid to how AI tools contributed to decision-making, speed, coordination, or patient outcomes. Repeated references across sections were used sparingly and only when the tool or dataset had multi-domain relevance.

Inclusion criteria

Studies were included if they were peer-reviewed, published in English, and focused on AI applications in the context of acute ischemic or hemorrhagic stroke. Eligible studies were either open access or accessible through institutional databases and reported clinical, operational, or system-level findings relevant to stroke care. Preference was given to articles that included performance metrics such as AUC, sensitivity, or diagnostic accuracy. Additionally, studies were required to be either U.S.-based or international in origin but with high applicability to the U.S. healthcare system.

Exclusion criteria

Exclusion criteria encompassed non-English publications, non-peer-reviewed content, and studies limited to algorithm development without clinical implementation. Opinion pieces, editorials, inaccessible full texts, duplicate entries, and papers lacking original data or outcome reporting were also excluded from the final synthesis.

The studies chosen for review

**Figure 1:**
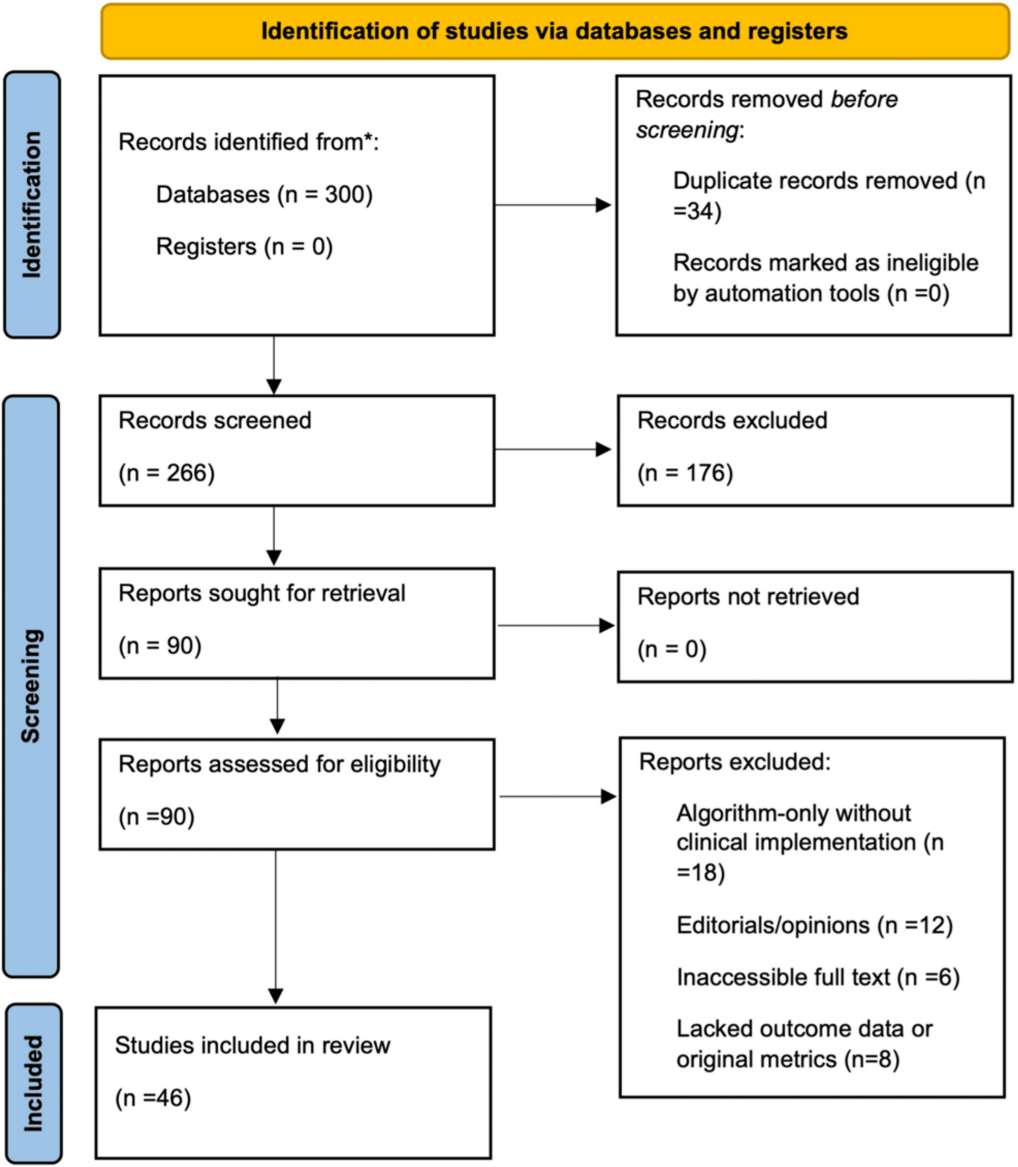
PRISMA Article Selection Flow Diagram PRISMA: Preferred Reporting Items for Systematic Reviews and Meta-Analyses.

The Preferred Reporting Items for Systematic Review and Meta-Analyses (PRISMA) flow diagram in Figure 1 outlines the process used to identify, screen, and include studies in this narrative review on AI applications in acute stroke care. A total of 300 records were identified through structured database searches, with 34 duplicates removed. Title and abstract screening were conducted on 266 records, followed by full-text review of 90 articles. After applying predefined inclusion and exclusion criteria, 46 studies were selected for final synthesis based on relevance, methodological rigor, and clinical applicability to U.S. stroke care settings.

A total of 46 studies were included in the final synthesis. While some were referenced in more than one thematic section, each was selected for its distinct contribution to the understanding of AI in stroke care across domains.

Table 1 provides a consolidated overview of selected peer-reviewed studies that assessed AI tools in the context of acute stroke care. Each entry is categorized by its primary domain of use: diagnostic imaging, predictive modeling, or workflow optimization. The studies included demonstrate measurable clinical or operational relevance, with a focus on functions such as image analysis, risk stratification, and care coordination. Performance outcomes are reported where available. These studies form the evidentiary basis for the core analysis presented in this review and were selected according to established inclusion criteria.

**Table 1:** AI Tools Referenced throughout Review Abbreviations: Viz.ai: Viz.ai stroke triage platform (Viz.ai, Inc., San Francisco, CA, USA); SIBILLA: Stroke Individualized outcome prediction with XGBoost–Based Machine Learning; AutoML: Automated Machine Learning; LVO: large-vessel occlusion; CTA: CT angiography; AUC: area under the curve; mRS: modified Rankin Scale; DWI: diffusion-weighted imaging; EVT: endovascular therapy; SHAP: SHapley Additive exPlanations; NIHSS: National Institutes of Health Stroke Scale.

AI Tool	Domain	Function	Key Metric	Reference
RapidAI	Diagnostic Imaging	Automated CT perfusion analysis to determine core infarct and penumbra volumes for EVT eligibility.	Sensitivity of 88% for large core infarct detection using Tmax >6s threshold; validated against DWI.	[17]
RapidAI	Workflow Optimization	Enabled rapid triage with automated imaging and alerting to reduce endovascular treatment delays.	Median time from CTA to intervention reduced by 20 minutes with AI-enabled workflow.	[31]
Viz.AI	Workflow Optimization	Used AI to detect LVO and push alerts to stroke teams, improving patient routing and transfer time.	Door-in-door-out time reduced by 66 minutes on average in transfer cases.	[27]
Viz.AI	Workflow Optimization	Integrated AI-powered triage and telemedicine into routine stroke response at primary centers.	Improved door-to-needle times and 2x increase in treatment rates within thrombolytic window.	[45]
SIBILLA (XGBoost- Based)	Predictive Modelling	Predicted 3-month functional outcome using clinical, lab, and imaging data with explainability via SHAP.	Prediction accuracy 78% for favorable mRS (0–2); validated on internal and external datasets.	[37]
AutoML	Predictive Modelling	Used ensemble ML to predict hemorrhagic transformation after ischemic stroke.	Best-performing AutoML model achieved AUC of 0.85 for predicting transformation risk.	[39]
SHAP	Predictive Modelling	Applied SHAP for model transparency, identifying key stroke predictors including glucose and NIHSS.	SHAP consistently ranked glucose and NIHSS as dominant contributors to 3-month outcome models.	[15]

Limitations and challenges of AI in stroke care

Despite significant progress in stroke-focused artificial intelligence, barriers remain that limit clinical reliability. Many AI models are trained on retrospective datasets from single centers, restricting generalizability to broader populations. Alaka et al. showed that machine learning models often outperform regression methods on internal data but underperform in external settings, raising concerns about overfitting and performance drift [35,36]. Heo et al. reported similar findings, where accuracy declined when predictive models were tested outside their derivation cohort [38]. These issues matter most in predictive tools, where calibration errors can alter risk thresholds, but diagnostic and workflow platforms are not immune. Bojsen et al. found considerable heterogeneity in diagnostic performance across vendors and imaging protocols, highlighting inconsistencies in LVO and MRI-based tools [20]. Workflow platforms such as those studied in the VALIDATE and Viz.ai implementations improved notification times, yet their effectiveness depended on alignment with local systems and teams [26,27].

A second challenge lies in the interpretability of models, which remains uneven across domains. Al-Janabi et al. observed that many AI systems provide accurate outputs without revealing their underlying logic, creating trust gaps at the point of care [18]. Alaka et al. emphasized that the opacity of black-box models is incompatible with time-sensitive stroke decisions [36]. Jabal et al. demonstrated that interpretable models can provide clinically meaningful explanations of feature importance, although transparency often trades off against predictive accuracy [40]. These findings suggest that explainability is not a secondary issue but a central requirement for clinical adoption. While techniques such as interpretable feature mapping are being applied, more advanced frameworks commonly cited in machine learning, including SHAP and LIME, remain underutilized in stroke AI [40].

Integration barriers also limit the operational value of otherwise effective tools. Devlin et al. reported that AI coordination platforms faced challenges in aligning with PACS, EMS, and electronic health record systems [26]. Figurelle et al. similarly showed that Viz.ai adoption improved workflow times but required sustained infrastructure support and coordination across departments [27]. In resource-limited or rural environments, Hassan et al. found that technical capacity and workforce familiarity reduced the ability to replicate improvements documented in tertiary centers [19]. Cost and resource requirements therefore remain major obstacles, and economic analyses of reimbursement or scalability are still largely absent.

Clinician workload presents another concern. Ali et al. highlighted that excessive or non-actionable alerts contribute to fatigue, which undermines both urgency and adoption [44]. Although alert fatigue has been qualitatively reported, it has not been systematically quantified in stroke AI trials. These operational issues underscore that effectiveness is shaped as much by system readiness as by algorithmic precision.

Equity, regulation, and medico-legal accountability represent further unresolved challenges. Al-Janabi et al. noted that many datasets exclude underrepresented populations and rare stroke phenotypes, embedding bias into outputs [18]. Broader disparities in stroke care, including socioeconomic and geographic gaps, mean that AI may inadvertently widen inequities if not carefully deployed [8,9,19]. On the regulatory front, Bluemke et al. criticized the absence of unified standards for radiology AI, noting the lack of clear oversight, liability, and approval pathways [34]. Wardlaw et al. added that governance gaps include limited transparency, inadequate data auditability, and unresolved privacy protections [22]. These shortcomings leave medico-legal risks ill-defined, particularly when AI errors influence acute stroke decisions where accountability must be immediate.

Overall, limitations differ across domains. Diagnostic tools suffer from vendor and protocol heterogeneity, predictive models from overfitting and drift, and workflow platforms from dependence on infrastructure and human coordination. These challenges will require stronger external validation, systematic approaches to interpretability, and frameworks that address regulatory, ethical, and equity concerns alongside technical refinement [18-20,22,26,27,34-36,38-40,44].

In summary, the most pressing barriers to AI adoption in stroke care are the lack of external validation across diverse populations, persistent challenges in workflow integration with hospital systems, and unresolved issues of model interpretability and accountability. Addressing these gaps will be essential for transitioning AI from promising innovation to dependable infrastructure within stroke systems of care.

Synthesis and future directions

AI's role in stroke care is converging across diagnostic, predictive, and workflow domains, automating high-frequency, time-sensitive, and data-heavy processes. In diagnostic imaging, one of the most widely used examples is Rapid CTA, an AI-powered software developed by RapidAI that analyzes CTA scans to rapidly identify and assess LVOs. The tool has achieved near real-time detection of critical findings and has been associated with expedited activation of stroke teams in high-acuity cases [31]. Predictive platforms such as those developed in the Stroke Individualized outcome prediction with XGBoost-Based Machine Learning (SIBILLA) project have shown accurate NIHSS estimation within 24 hours of admission, supporting early risk stratification and planning [37]. Across all domains, AI functions as a supportive scaffold that strengthens decision speed and consistency. It reinforces multidisciplinary coordination rather than replacing clinical reasoning, and it supports timely action without undermining physician autonomy.

The current evidence base illustrates both progress and persistent limitations. For LVO detection, most evaluations remain retrospective, with reported sensitivities between 80% and 96% and specificities of 90%-98% across vendor platforms [22]. By contrast, predictive models such as SIBILLA were tested prospectively in clinical cohorts [37], and several outcome-prediction tools have undergone external validation, though they demonstrated poorer calibration than traditional regression models [35]. Calibration problems matter clinically because overestimation or underestimation of risk can distort treatment thresholds and reduce dependability at the bedside. Transparency also remains uneven. Interpretable machine learning strategies now provide clinically meaningful explanations of feature importance, offering one avenue for strengthening trust and clinician engagement [40].

Beyond performance, broader system considerations define the future of AI adoption. Most published validations still occur under idealized or simulated conditions rather than the heterogeneous realities of daily practice [38]. The absence of large, multicenter studies in varied environments, including underserved or resource-limited hospitals, limits confidence in generalizability [40]. Evidence standards for diagnostic AI tools remain inconsistent, as they are not subjected to the same regulatory pathways as therapeutics, leaving governance and approval mechanisms underdeveloped [22]. Long-term adaptation must therefore be understood as more than algorithm design. It encompasses monitoring and retraining of deployed models, infrastructure alignment with PACS, EHRs, and telestroke networks, as well as workflow redesign to sustain improvements over time [22,31,35,40]. Cost-effectiveness, reimbursement, and scalability have yet to be adequately tested, but the observed reductions in treatment delays and the persistent gaps in stroke access across high- and low-resource settings highlight the urgency of these questions [6,8,22,31,37]. Future progress will also require interdisciplinary collaboration among clinicians, engineers, ethicists, and policymakers to ensure that advances in accuracy and efficiency translate into trustworthy, equitable, and sustainable patient care [22,40].

## Conclusions

This narrative review examined the evolving role of artificial intelligence in stroke care, spanning diagnostic imaging, predictive modeling, and workflow optimization. Across these domains, AI demonstrates meaningful contributions in enhancing speed, reducing variability, and supporting decision-making processes under intense clinical pressure. Rather than functioning as a clinical replacement, AI serves as an operational adjunct, reinforcing existing systems by processing high-volume data, triggering coordinated responses, and aiding in outcome forecasting with improved consistency.

However, AI's potential is shaped not solely by algorithmic capability but by the readiness of the environments into which it is deployed. The literature reveals ongoing challenges related to external validation, workflow integration, clinician trust, and ethical design. These limitations underscore the importance of framing AI not as a standalone solution, but as part of a broader infrastructure strategy grounded in interoperability, transparency, and equity. Moving forward, the impact of AI in stroke care will be defined less by abstract technological milestones and more by tangible demonstrations of clinical benefit. For instance, Rapid CTA has achieved a high positive predictive value for large-vessel occlusion at a 45% relative vessel density threshold, enabling active worklist reprioritization. Its deployment has been associated with shorter treatment times and improved outcomes, underscoring how targeted innovations can reshape care pathways in measurable ways. Ultimately, the promise of AI in stroke care will be realized not through novelty alone, but through its capacity to consistently deliver safer, faster, and more equitable outcomes for patients.

While current evidence reflects both proof-of-concept models and real-world deployments, the trajectory of AI in stroke care will depend on broader validation and system-level adoption. Future research should prioritize large multicenter trials that evaluate generalizability across diverse populations and care settings, alongside equity-focused studies that address disparities in access and outcomes. Sustainability will also hinge on cost-effectiveness analyses and the ability to scale solutions across both high-resource and low-resource health systems. Equally important, regulatory and policy frameworks must evolve in parallel to ensure responsible integration, safeguard patient trust, and clarify the evolving roles of stroke teams within AI-enabled pathways.

## References

[1] Pu L, Wang L, Zhang R, Zhao T, Jiang Y, Han L (2023). Projected global trends in ischemic stroke incidence, deaths and disability-adjusted life years from 2020 to 2030. Stroke.

[2] Renedo D, Acosta JN, Leasure AC (2024). Burden of ischemic and hemorrhagic stroke across the US from 1990 to 2019. JAMA Neurol.

[3] Leasure AC, Acosta J, Sharma R, Krumholz HM, Havenon A, Falcone GJ, Sheth KN (2022). Abstract 103: burden of ischemic and hemorrhagic stroke across the US from 1990-2019: a Global Burden of Disease study. Stroke.

[4] Pulvers JN, Watson JD (2017). If time is brain where is the improvement in prehospital time after stroke?. Front Neurol.

[5] Yperzeele L, Van Hooff RJ, De Smedt A (2014). Prehospital stroke care: limitations of current interventions and focus on new developments. Cerebrovasc Dis.

[6] Saini V, Guada L, Yavagal DR (2021). Global epidemiology of stroke and access to acute ischemic stroke interventions. Neurology.

[7] Perna R, Temple J (2015). Rehabilitation outcomes: ischemic versus hemorrhagic strokes. Behav Neurol.

[8] Feigin VL, Brainin M, Norrving B (2022). World Stroke Organization (WSO): Global Stroke Fact Sheet 2022. Int J Stroke.

[9] Morgenstern LB, Kissela BM (2015). Stroke disparities: large global problem that must be addressed. Stroke.

[10] Paolucci S, Antonucci G, Grasso MG (2003). Functional outcome of ischemic and hemorrhagic stroke patients after inpatient rehabilitation: a matched comparison. Stroke.

[11] Furie KL, Jayaraman MV (2018). 2018 guidelines for the early management of patients with acute ischemic stroke. Stroke.

[12] Lachkhem Y, Rican S, Minvielle É (2018). Understanding delays in acute stroke care: a systematic review of reviews. Eur J Public Health.

[13] Akbik F, Xu H, Xian Y (2020). Trends in reperfusion therapy for in-hospital ischemic stroke in the endovascular therapy era. JAMA Neurol.

[14] Kamal N, Sheng S, Xian Y (2017). Delays in door-to-needle times and their impact on treatment time and outcomes in get with the guidelines - stroke. Stroke.

[15] Hayes M, Schlundt D, Bonnet K, Vogus TJ, Kripalani S, Froehler MT, Ward MJ (2019). Tales from the trips: a qualitative study of timely recognition, treatment, and transfer of emergency department patients with acute ischemic stroke. J Stroke Cerebrovasc Dis.

[16] Lima A, Sapkota S, Auchus AP, Sugg R (2012). Abstract 69: does advanced imaging delay time to administration of intravenous rt-PA in the acute ischemic stroke patient?. Stroke.

[17] Akay EM, Hilbert A, Carlisle BG, Madai VI, Mutke MA, Frey D (2023). Artificial intelligence for clinical decision support in acute ischemic stroke: a systematic review. Stroke.

[18] Al-Janabi OM, El Refaei A, Elgazzar T (2024). Current stroke solutions using artificial intelligence: a review of the literature. Brain Sci.

[19] Hassan AE, Ringheanu VM, Rabah RR, Preston L, Tekle WG, Qureshi AI (2020). Early experience utilizing artificial intelligence shows significant reduction in transfer times and length of stay in a hub and spoke model. Interv Neuroradiol.

[20] Bojsen JA, Elhakim MT, Graumann O (2024). Artificial intelligence for MRI stroke detection: a systematic review and meta-analysis. Insights Imaging.

[21] Hillis JM, Bizzo BC, Gauriau R (2023). Enhanced physician performance when using an artificial intelligence model to detect ischemic stroke on computed tomography. medRxiv Neurology.

[22] Wardlaw JM, Mair G, von Kummer R (2022). Accuracy of automated computer-aided diagnosis for stroke imaging: a critical evaluation of current evidence. Stroke.

[23] Amukotuwa SA, Straka M, Smith H, Chandra RV, Dehkharghani S, Fischbein NJ, Bammer R (2019). Automated detection of intracranial large vessel occlusions on computed tomography angiography: a single center experience. Stroke.

[24] Gibson E, Georgescu B, Ceccaldi P (2022). Artificial intelligence with statistical confidence scores for detection of acute or subacute hemorrhage on noncontrast CT head scans. Radiol Artif Intell.

[25] Kiththiworaphongkich W, Khamwongsa N, Chaimongkol P (2024). Reliability and radiologists’ concordance of AI-calculated Alberta Stroke Program Early CT Score (ASPECTS). ASEAN Journal of Radiology.

[26] Devlin T, Gao L, Collins O (2024). VALIDATE - utilization of the Viz.ai mobile stroke care coordination platform to limit delays. Frontiers in Stroke.

[27] Figurelle ME, Meyer DM, Perrinez ES (2023). Viz.ai Implementation of stroke augmented intelligence and communications platform to improve indicators and outcomes for a comprehensive stroke center and network. AJNR Am J Neuroradiol.

[28] Chin V, Yeboah K, Balushi A, Guthri A, Christopher K, Edgell R (2020). E-068 Improving efficiency of acute ischemic stroke therapies: reducing door-to-needle and door-to-puncture time. Journal of NeuroInterventional Surgery.

[29] Field NC, Entezami P, Boulos AS, Dalfino J, Paul AR (2023). Artificial intelligence improves transfer times and ischemic stroke workflow metrics. Interv Neuroradiol.

[30] Frost E, Penckofer M, Zhang L (2024). Door to puncture in large vessel occlusions pre‐ and post-AI implementation. Stroke: Vascular and Interventional Neurology.

[31] Yahav-Dovrat A, Saban M, Merhav G (2021). Evaluation of artificial intelligence-powered identification of large-vessel occlusions in a comprehensive stroke center. AJNR Am J Neuroradiol.

[32] Delio PR, Wong ML, Tsai JP (2021). Assistance from automated ASPECTS software improves reader performance. J Stroke Cerebrovasc Dis.

[33] Gupta AC, Schaefer PW, Chaudhry ZA (2012). Interobserver reliability of baseline noncontrast CT Alberta Stroke Program Early CT Score for intra-arterial stroke treatment selection. AJNR Am J Neuroradiol.

[34] Bluemke DA, Moy L, Bredella MA (2020). Assessing radiology research on artificial intelligence: a brief guide for authors, reviewers, and readers - from the Radiology Editorial Board. Radiology.

[35] Alaka SA, Menon BK, Brobbey A (2020). Functional outcome prediction in ischemic stroke: a comparison of machine learning algorithms and regression models. Front Neurol.

[36] Fast L, Temuulen U, Villringer K (2023). Machine learning-based prediction of clinical outcomes after first-ever ischemic stroke. Front Neurol.

[37] Caliandro P, Lenkowicz J, Reale G (2024). Artificial intelligence to predict individualized outcome of acute ischemic stroke patients: the SIBILLA project. Eur Stroke J.

[38] Heo J, Yoon JG, Park H, Kim YD, Nam HS, Heo JH (2019). Machine learning-based model for prediction of outcomes in acute stroke. Stroke.

[39] Jiang YL, Zhao QS, Li A, Wu ZB, Liu LL, Lin F, Li YF (2024). Advanced machine learning models for predicting post-thrombolysis hemorrhagic transformation in acute ischemic stroke patients: a systematic review and meta-analysis. Clin Appl Thromb Hemost.

[40] Jabal MS, Joly O, Kallmes D, Harston G, Rabinstein A, Huynh T, Brinjikji W (2022). Interpretable machine learning modeling for ischemic stroke outcome prediction. Front Neurol.

[41] Jang SK, Chang JY, Lee JS (2020). Reliability and clinical utility of machine learning to predict stroke prognosis: comparison with logistic regression. J Stroke.

[42] Bivard A, Levi C, Lin L (2017). Validating a predictive model of acute advanced imaging biomarkers in ischemic stroke. Stroke.

[43] Grech R, Galvin PL, Power S, O'Hare A, Looby S, Brennan P, Thornton J (2014). Outcome prediction in acute stroke patients considered for endovascular treatment: a novel tool. Interv Neuroradiol.

[44] Ali F, Hamid U, Zaidat O, Bhatti D, Kalia JS (2020). Role of artificial intelligence in TeleStroke: an overview. Front Neurol.

[45] Gunda B, Neuhaus A, Sipos I (2022). Improved stroke care in a primary stroke centre using AI-decision support. Cerebrovasc Dis Extra.

[46] Yaeger KA, Shoirah H, Kellner CP, Fifi J, Mocco J (2019). Emerging technologies in optimizing pre-intervention workflow for acute stroke. Neurosurgery.

